# Sex Differences in Seasonal Variation in Metabolic Syndrome and Its Components: A 10-Year National Health Screening Study

**DOI:** 10.3390/jcm14175968

**Published:** 2025-08-23

**Authors:** Hyun-Sun Kim, Hyun-Jin Kim, Dongwoo Kang, Jungkuk Lee

**Affiliations:** 1 Department of Nursing, College of Nursing, Eulji University, Uijeongbu 11759, Republic of Korea; hs.kim@eulji.ac.kr; 2 Division of Cardiology, Department of Internal Medicine, Hanyang University Guri Hospital, Hanyang University College of Medicine, Guri 11923, Republic of Korea; 3 Department of Data Science, Hanmi Pharmaceutical Co., Ltd., Wiryeseong-daero, Songpa-gu, Seoul 05545, Republic of Korea; dongwoo.kang94@hanmi.co.kr (D.K.); jungkuk.lee@hanmi.co.kr (J.L.)

**Keywords:** metabolic syndrome, seasons, prevalence, sex factors

## Abstract

**Background/Objectives**: Metabolic syndrome (MetS) comprises a cluster of cardiometabolic risk factors that vary dynamically under environmental and behavioral influences. Although there are data suggesting seasonal variability in individual metabolic components, few studies have comprehensively assessed MetS as a composite condition across seasons using a large, nationally representative population. In this study, we aimed to evaluate the seasonal and monthly patterns of MetS prevalence and component burden, with a focus on sex-specific differences. **Methods**: We analyzed 5,507,251 health screening records from 2,057,897 Korean adults aged ≥40 years between 2013 and 2022, obtained from the National Health Insurance Service database. Seasons were categorized as: spring (March–May), summer (June–August), fall (September–November), and winter (December–February). Trends in MetS prevalence and its components were evaluated monthly and seasonally, stratified by sex. **Results**: MetS prevalence significantly varied by season in both sexes (*p* < 0.001), ranging from 30.2% to 34.5% in men and from 21.5% to 25.5% in women. Among men, a U-shaped pattern was observed, with the lowest prevalence during summer and a progressive increase through winter. Women showed a steady decline in prevalence from January to September, followed by a slight rebound. Winter was associated with increased odds of MetS in both sexes. A significant interaction between sex and season (p for interaction < 0.001) indicated the presence of sex-specific temporal patterns. **Conclusions**: This nationwide study revealed clear seasonal variation in MetS prevalence and component burden, with sex-specific patterns. These findings highlight the importance of incorporating seasonality and sex in cardiometabolic risk assessments and public health interventions.

## 1. Introduction

Metabolic syndrome (MetS) comprises a constellation of interrelated metabolic abnormalities, including central obesity, elevated blood pressure (BP), dyslipidemia, and impaired glucose regulation, which predispose individuals to cardiovascular diseases and type 2 diabetes [1]. As a major public health concern, MetS affects a substantial proportion of middle-aged and older adults and contributes to the global burden of cardiometabolic disease [2]. In addition to conventional risk factors, increasing attention has been directed toward the effect of seasonal variation on metabolic health. Previous studies have shown that physiological parameters, such as BP, lipid levels, and body weight, exhibit seasonal rhythms, with BP, triglyceride (TG) levels, waist circumference, and fasting glucose generally higher and high-density lipoprotein cholesterol (HDL-C) lower during colder months, presenting generally less favorable profiles during colder months [3,4,5,6,7,8]. These seasonal changes likely result from complex interactions among ambient temperature, daylight exposure, physical activity, and dietary behaviors [9,10]. However, most prior research has either focused on individual metabolic components or has been limited by small sample sizes and lack of population representativeness. Moreover, limited evidence exists on how seasonal effects on MetS may differ by sex. Men and women demonstrate different cardiometabolic phenotypes, behavioral patterns, and hormonal environments, which may influence the seasonal expression of MetS [11,12,13]. Understanding these sex-specific responses to seasonal variation is essential for developing targeted interventions and refining risk stratification. In this study, we aimed to evaluate the seasonal and monthly patterns of MetS prevalence and component burden, with particular attention to potential differences between men and women.

## 2. Materials and Methods

### 2.1. Study Design and Patient Population

**Figure 1:**
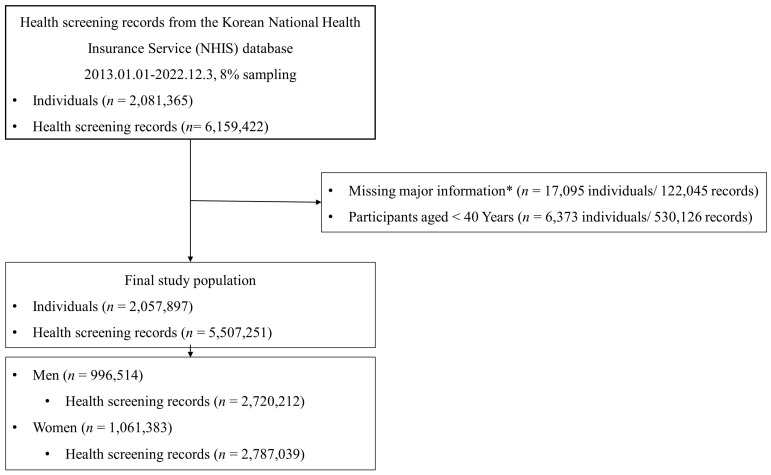
Study flowchart. * Height, weight, body mass index, systolic blood pressure, waist circumference, diastolic blood pressure, hemoglobin, fasting glucose, total cholesterol, Aspartate Aminotransferase, Alanine Transaminase, Triglyceride, low-density lipoprotein cholesterol, high-density lipoprotein cholesterol, creatinine, glomerular filtration rate, smoking status, alcohol consumption, and physical activity.

This retrospective observational study used data from the Korean National Health Insurance Service (NHIS) database (approval number: NHIS-2024-02-387). The database covers approximately 97% of the Korean population and provides comprehensive longitudinal information on demographics, diagnoses, prescriptions, health screenings, and healthcare utilization. The National Health Screening Program is conducted biennially for adults aged 40 years and older. From the NHIS database, an 8% random sample of adults who underwent at least one health screening between 1 January 2013 and 31 December 2022 was extracted. An initial total of 2,081,365 individuals with 6,159,422 screening records were identified. Individuals with missing essential variables–including height, weight, body mass index (BMI), systolic BP, waist circumference, diastolic BP, hemoglobin, fasting glucose, total cholesterol, aspartate aminotransferase, alanine transaminase, TG, low-density lipoprotein cholesterol (LDL-C), HDL-C, creatinine, estimated glomerular filtration rate, smoking status, alcohol consumption, or physical activity (*n* = 17,095; 122,045 records) and those younger than 40 years (*n* = 6373; 530,126 records) were excluded. The final study population included 2,057,897 individuals with 5,507,251 screening records (Figure 1). For sex-stratified analyses, the population was divided into men (*n* = 996,514; 2,720,212 records) and women (*n* = 1,061,383; 2,787,039 records) subgroups. Seasons were categorized based on the month of the health checkup: spring (March–May), summer (June–August), fall (September–November), and winter (December–February). This study was approved by the Institutional Review Board of Hanyang University Guri Hospital (IRB approval no. GURI 2024-02-018). The requirement for informed consent was waived because the NHID had previously obtained participant consent, and all data had been anonymized.

### 2.2. Data Collection

Demographic and clinical data were obtained from the NHIS Health Screening Database. Variables included age, sex, and household income level (categorized into quartiles based on the NHIS premium index), and residential region (classified as capital, metropolitan, or provincial areas). Anthropometric measurements included height, weight, BMI, waist circumference, and systolic and diastolic BP. BMI was categorized according to Asian-specific criteria as underweight (<18.5 kg/m^2^), normal weight (18.5–22.9 kg/m^2^), overweight (23.0–24.9 kg/m^2^), or obese (≥25.0 kg/m^2^) [14]. Lifestyle variables (smoking status, alcohol consumption, and physical activity) were assessed using the standardized self-reported questionnaire administered during the Korean NHIS health screening program. Participants were classified based on smoking status into non-smokers, past smokers, or current smokers. Current smoking was defined as having smoked at least 100 cigarettes and continuing to smoke at the time of screening [15,16]. High-risk alcohol consumption was defined as drinking on ≥2 days per week and consuming ≥7 standard drinks per occasion for men or ≥5 for women [16]. Regular physical activity refers to moderate-to-vigorous exercise performed at least five times per week for a minimum of 30 min per session [17]. Laboratory tests included measurements of fasting plasma glucose, TG, HDL-C, LDL-C, total cholesterol, and serum creatinine levels, which were measured using a standardized enzymatic method, as well as estimated glomerular filtration rate, calculated using the Modification of Diet in Renal Disease equation [18] adopted by the NHIS during the study period. All biochemical parameters were obtained after an overnight fast of at least 8 h, in accordance with the standardized NHIS health screening protocol. Data on comorbidities and medication use, including antihypertensives, glucose-lowering agents, lipid-lowering agents, and antiplatelet drugs, were collected using ICD-10 diagnostic codes and prescription records. Detailed definitions are provided in Supplementary Table S1.

### 2.3. Definition and Metabolic Syndrome

MetS was defined using harmonized international criteria, incorporating guidelines from the International Diabetes Federation; American Heart Association; National Heart, Lung, and Blood Institute; and others. Waist circumference cutoffs recommended by the Korean Society for the Study of Obesity were applied [1,14,16,19]. Participants were classified as having MetS if they met ≥3 of the following five criteria: (1) abdominal obesity (waist circumference ≥ 90 cm in men or ≥85 cm in women), (2) elevated TG levels (≥150 mg/dL), (3) low HDL-C levels (<40 mg/dL in men or <50 mg/dL in women), (4) elevated BP (systolic ≥ 130 mmHg, diastolic ≥ 85 mmHg, or use of antihypertensive medication), and (5) elevated fasting glucose levels (≥100 mg/dL or use of glucose-lowering medication). This definition was applied uniformly to all participants, including those with diabetes mellitus or cardiovascular disease, in accordance with the established guidelines. These criteria were uniformly applied across all seasons and stratified by sex.

### 2.4. Statistical Analysis

All analyses were conducted separately for men and women. Baseline characteristics were compared across seasons using analysis of variance for continuous variables and chi-square tests for categorical variables. The seasonal and monthly prevalence of MetS and its components were visualized using descriptive plots. For logistic regression analyses, only one representative health screening record per participant was included to satisfy the independence assumption. Specifically, the latest available health screening record during the study period (2013–2022) was selected for each participant. To evaluate the association between season and MetS prevalence, multivariate logistic regression models were developed for each sex, using spring as the reference category. Model 1 was adjusted for age; model 2 additionally included lifestyle variables (smoking status, alcohol consumption, and physical activity); and model 3 was adjusted for socioeconomic factors (household income and residential region). Odds ratios (ORs) and 95% confidence intervals (CIs) were also determined. Forest plots were generated to visually present the ORs and 95% CI from the fully adjusted logistic regression models (Model 3) for the association between season and metabolic syndrome. The forest plots were created separately by sex, with spring as the reference category. To assess linear seasonal trends, the *p*-value for trend was computed by modeling the four seasons as an ordinal variable. Trend analyses were performed separately by sex. To determine whether seasonal trends differed significantly between sexes, an interaction term (sex × season) was included in the fully adjusted model. A significant interaction indicated a sex-specific effect of season on MetS prevalence. Statistical significance was defined as a two-sided *p*-value < 0.05. All analyses were performed using SAS 9.4 (SAS Institute Inc., Cary, NC, USA).

## 3. Results

### 3.1. Baseline Characteristics

A total of 5,507,251 health screening records were analyzed, including 2,720,212 records from men and 2,787,039 records from women. Seasonal variation was evident across multiple demographic, clinical, socioeconomic, and lifestyle factors in both sexes (all *p* < 0.0001 unless otherwise noted) (Table 1 and Table 2). Among men (Table 1), those screened in spring were on average older, with the highest proportion of individuals aged 65 years or older (25.2%). During winter, men exhibited the highest mean values for systolic and diastolic BP, waist circumference, and fasting glucose levels. Obesity (BMI ≥ 25.0 kg/m^2^) was most prevalent in fall and winter (43.6% and 45.2%, respectively), whereas high-risk alcohol consumption and current smoking were reported most frequently during winter. Regular physical activity showed the lowest prevalence in winter (23.4%), with higher rates observed in other seasons. Women displayed similar seasonal patterns (Table 2). Those screened in spring were the oldest, with the highest proportion aged 65 years or older (31.4%). Winter examinations showed peak mean values for systolic and diastolic BP, waist circumference, and fasting glucose. The highest obesity prevalence occurred in winter (35.9%), coinciding with the lowest reported levels of regular exercise (21.6%) and highest prevalence of high-risk drinking. Additionally, HDL-C levels were highest in fall (60.6 ± 15.2 mg/dL) and lowest in summer (58.9 ± 15.7 mg/dL), whereas TG levels peaked in winter. The prevalence of hypertension, diabetes, and dyslipidemia was highest in spring, along with the corresponding use of antihypertensive, glucose-lowering, and lipid-lowering medications in both sexes.

### 3.2. Seasonal and Monthly Trends in Metabolic Syndrome Prevalence by Sex

**Figure 2:**
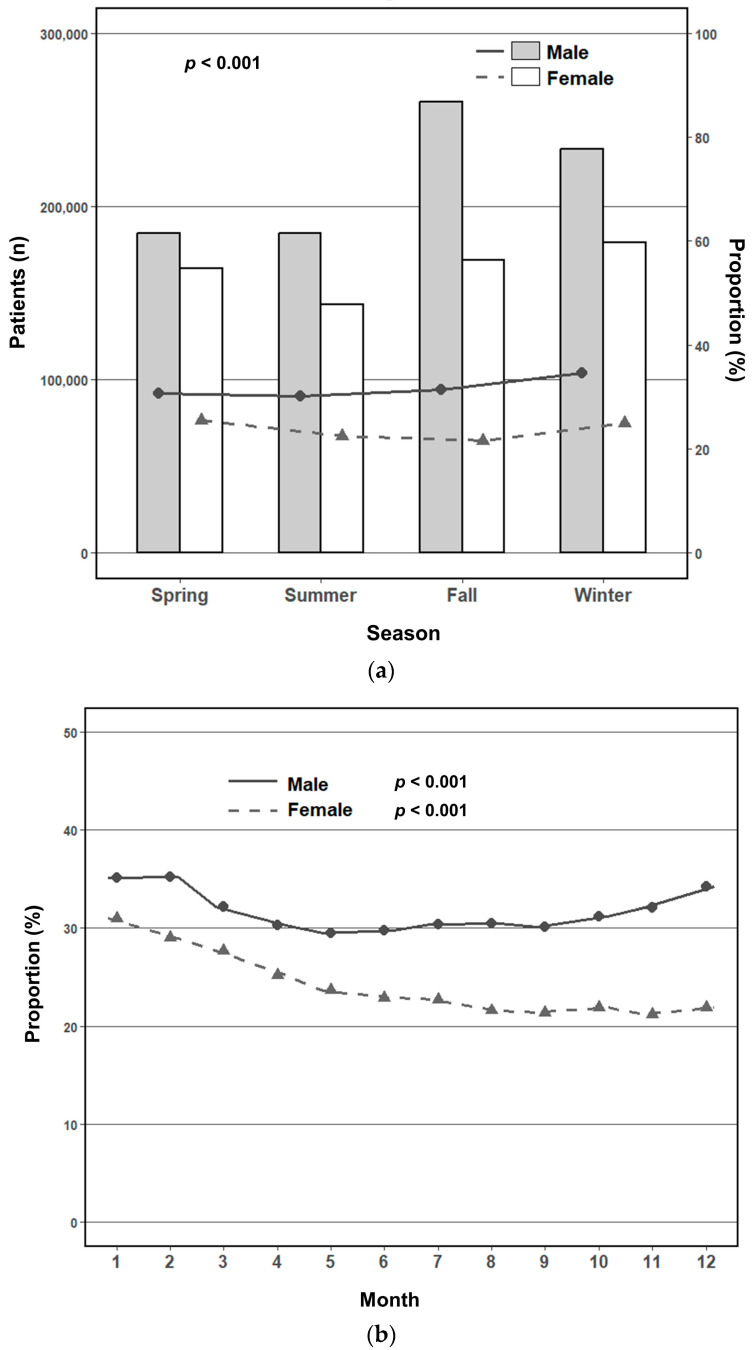
Prevalence of metabolic syndrome by sex: (**a**) seasonal variation in the prevalence of metabolic syndrome; (**b**) monthly variation in the prevalence of metabolic syndrome.

**Figure 3:**
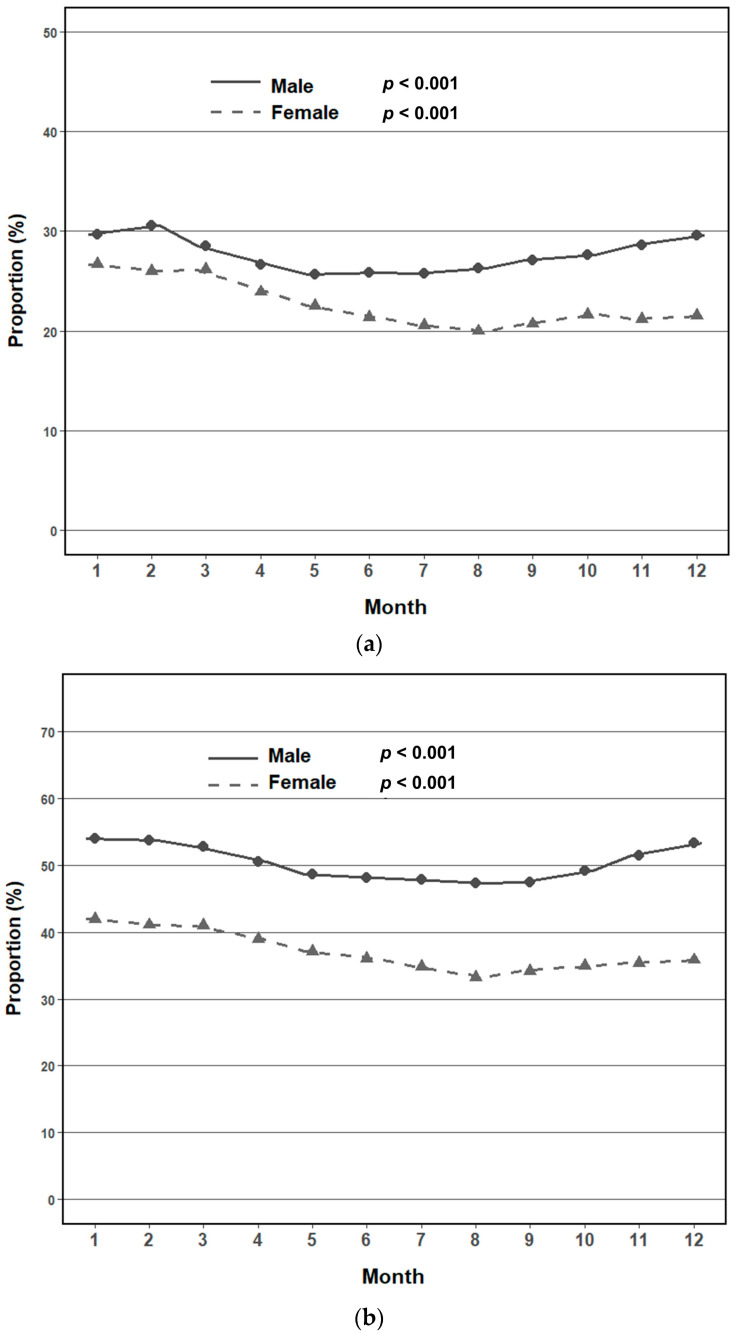

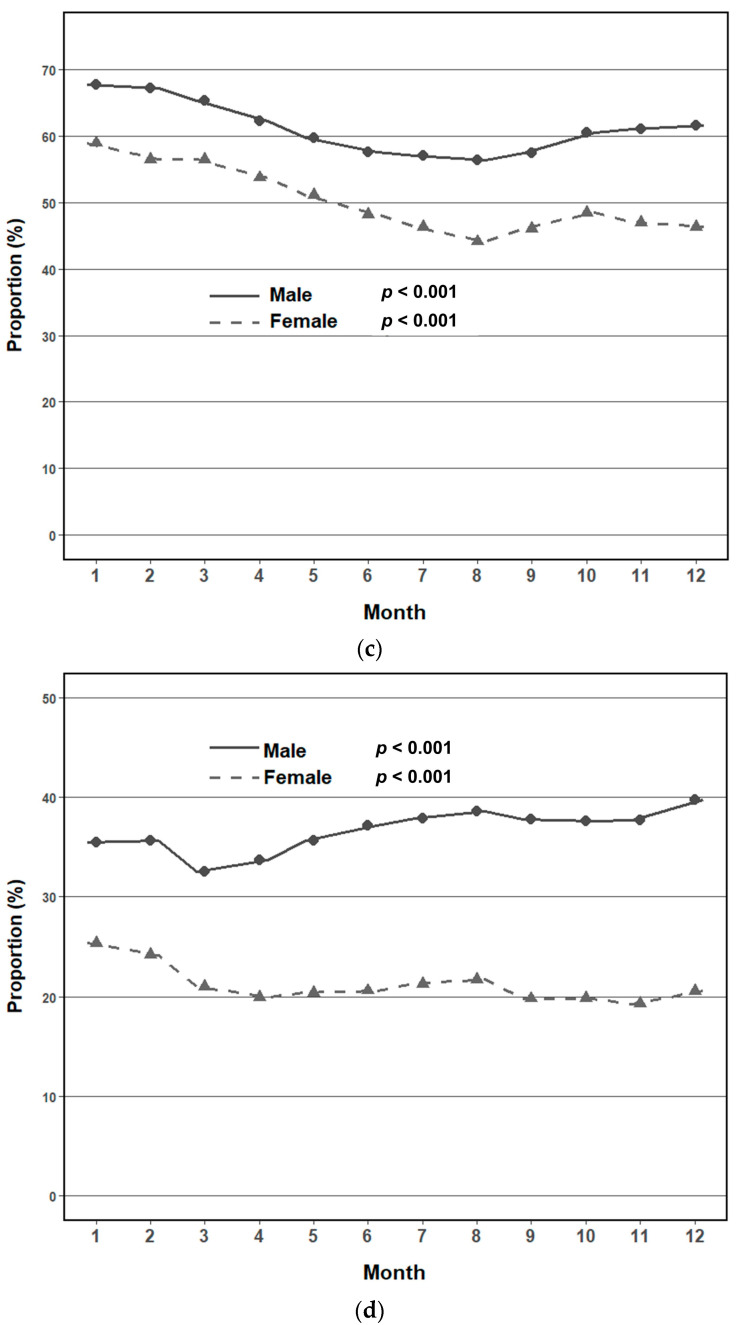

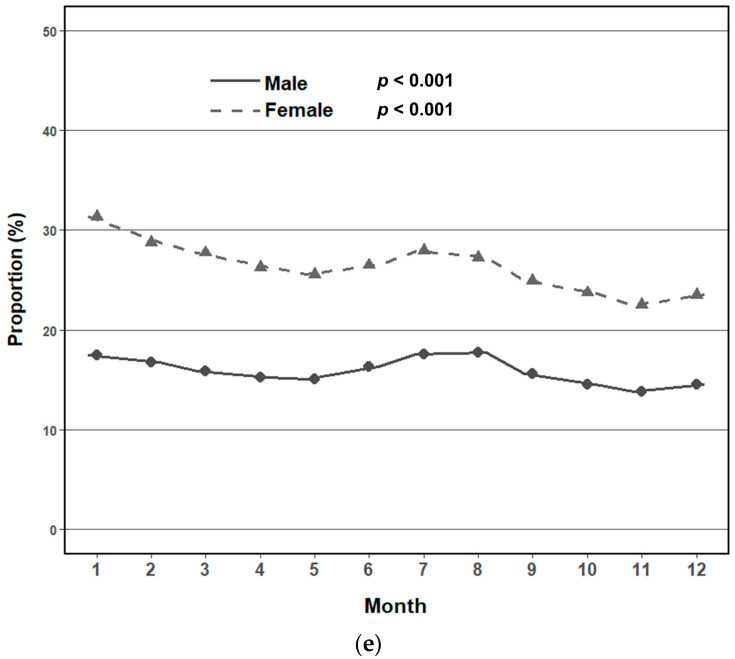
Prevalence of metabolic syndrome components by sex: (**a**) monthly variation in the prevalence of abdominal obesity; (**b**) monthly variation in the prevalence of elevated fasting glucose levels; (**c**) monthly variation in the prevalence of elevated blood pressure; (**d**) monthly variation in the prevalence of hypertriglyceridemia; (**e**) monthly variation in the prevalence of low high-density lipoprotein cholesterolemia.

The prevalence of MetS exhibited marked seasonal variation in both sexes (*p* < 0.0001 for all comparisons) (Table 3 and Figure 2a). Among men, the prevalence peaked in fall (31.3%) and winter (34.5%), with lower rates observed in spring (30.6%) and summer (30.2%). In women, the highest prevalence occurred in spring (25.5%) and decreased progressively through fall (21.5%) before rising again in winter (24.9%). Monthly analyses revealed distinct sex-specific trends (Figure 2b). In men, prevalence gradually declined from January to June and subsequently increased through December (*p* for trend < 0.001). By contrast, women exhibited a consistent decline from January to September, followed by a modest rebound from October to December (*p* for trend < 0.001). The difference in monthly trajectories between sexes was statistically significant (*p* for interaction < 0.001), indicating sex-specific seasonal patterns in metabolic risk. Seasonal distribution of the MetS components also differed according to sex (Table 3). In men, the proportion of individuals presenting with three or more components reached its highest levels during fall and winter. Most metabolic components were more prevalent in winter than in other seasons, except for low HDL cholesterol levels. In women, the burden of three or more components was greatest in spring and winter, with peak prevalence of most components occurring in spring, except for hypertriglyceridemia and low HDL cholesterol. Monthly trends in individual metabolic components further supported these seasonal patterns (Figure 3a–e). Elevated waist circumference, high fasting glucose levels, and high BP typically reached their lowest levels in summer, followed by a gradual increase toward late fall or winter. Hypertriglyceridemia showed a distinct nadir in early spring (March), with a subsequent increase during the latter months of the year. By contrast, low HDL cholesterol levels demonstrated a unique seasonal pattern, with the highest prevalence occurring during summer, particularly among women.

### 3.3. Adjusted Seasonal Association with Metabolic Syndrome by Sex

**Figure 4:**
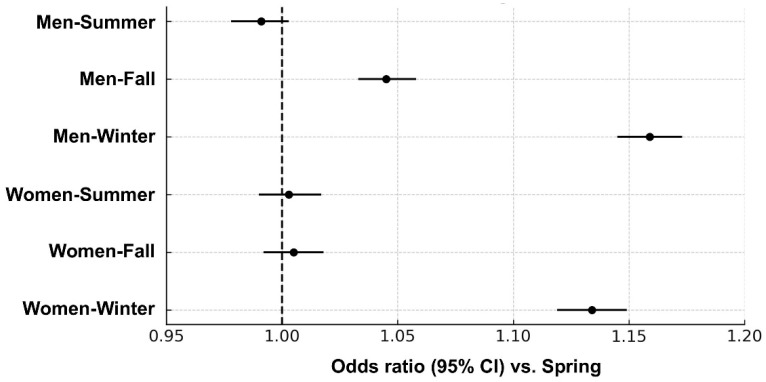
Forest plot for the association between season and metabolic syndrome, by sex. Spring as the reference category (Model 3). Odds ratios are adjusted for age, smoking status, alcohol consumption, physical activity, household income, and area of residence. CI, confidence interval.

Supplementary Table S2 summarizes the results of multivariate logistic regression analyses evaluating the seasonal association with MetS prevalence, using spring as the reference category. In men, after adjusting for age, lifestyle, and socioeconomic factors (model 3), fall and winter were significantly associated with increased odds of MetS (Figure 4). The adjusted ORs were 1.159 (95% CI, 1.145–1.173; *p* < 0.0001) for winter and 1.045 (95% CI, 1.033–1.058; *p* < 0.0001) for fall, indicating a sustained elevation in risk compared with spring. A non-significant trend toward lower risk was observed in summer. Seasonal patterns differed in women. Although unadjusted analyses revealed lower odds of MetS in all other seasons compared with spring, these associations were attenuated after full adjustment (Supplementary Table S2). In the fully adjusted model (model 3), only winter remained significantly associated with increased odds of MetS (OR: 1.134; 95% CI: 1.119–1.149; *p* < 0.0001), whereas associations for fall and summer lost statistical significance (Figure 4). Additional analyses evaluating the number of MetS components revealed that winter was consistently associated with increased odds of a greater component burden in both sexes. Conversely, summer was associated with lower odds of having ≥1 or ≥2 components, although these protective effects were modest after full adjustment.

## 4. Discussion

In this large population-based cohort study involving over 5.5 million health screening records, significant seasonal variation was observed in the prevalence of MetS and its components among both men and women. The prevalence of MetS peaked in winter and reached its lowest point in summer in men, and peaked in spring and was lowest in fall in women. Monthly trend analyses revealed a distinct U-shaped pattern in men, with prevalence declining from January to June and increasing thereafter. By contrast, women showed a gradual decline in prevalence until September, followed by a modest rebound in later months. These seasonal patterns remained statistically significant after adjusting for potential confounders, with winter being consistently associated with higher odds of MetS in both sexes. The burden of MetS components followed similar seasonal trajectories, typically peaking during colder months and reaching nadir during warmer seasons. Notably, sex-specific differences emerged in these trends, with a statistically significant interaction between sex and season affecting MetS prevalence.

Several physiological and behavioral factors may underlie the observed seasonal variation in MetS prevalence. Cold temperatures during fall and winter are known to increase sympathetic activity, promote vasoconstriction, and elevate BP, contributing to a higher incidence of hypertension, a key component of MetS [20,21]. Reduced daylight exposure, particularly during winter, may impair circadian regulation and decrease physical activity levels [22,23]. Seasonal dietary changes, such as increased caloric and fat intake in colder months, may also contribute to weight gain, dyslipidemia, and hyperglycemia [24,25,26]. By contrast, summer is often associated with increased physical activity, greater sunlight exposure, and improved glycemic control, potentially leading to a lower prevalence of MetS and its components [27,28]. These seasonal shifts likely interact in a complex manner to influence the expression of metabolic risk throughout the year.

Although the overall seasonal trend of higher MetS prevalence during colder months was consistent across sexes, distinct monthly patterns emerged between men and women. Specifically, men exhibited a U-shaped curve, with the lowest prevalence occurring mid-year, whereas women showed a steady decline from January to September, followed by a modest rebound toward the year’s end. This sex-based difference in temporal dynamics may reflect distinct regulatory patterns rather than stark contrasts in absolute metabolic risk. A plausible explanation involves the differential responsiveness of metabolic parameters to environmental fluctuations; in men, these parameters may respond more acutely to short-term changes in ambient temperature and photoperiod [29,30]. By contrast, in women, behavioral and hormonal factors may exert a more sustained, cumulative effect throughout the year. Estrogen, for example, generally enhances insulin sensitivity, improves lipid metabolism, and promotes vascular function. Estrogen decline during menopause may therefore adversely affect these parameters, contributing to increased central adiposity, higher TG levels, lower HDL-C, elevated BP, and higher fasting glucose, which together exacerbate the overall metabolic risk profile in postmenopausal women. Fluctuations in estrogen levels, especially among pre- and perimenopausal women, could contribute to a smoother, less abrupt seasonal pattern [31,32]. Furthermore, women may possess a greater internal regulatory stability against external environmental stimuli, leading to a better buffered response to acute seasonal stress [33]. Taken together, these findings suggest that seasonal variation in cardiometabolic risk is shaped by sex-specific weighting of environmental, behavioral, and hormonal influences.

Previous studies have suggested seasonal variability in individual metabolic components, such as BP, lipid profiles, and glucose metabolism; however, few have comprehensively assessed MetS as a composite condition across seasons using a large, nationally representative population [5,6,34,35,36,37]. A recent large-scale study using data from the Specific Health Checkups in the Kokuho Database in Osaka, Japan, examined seasonal changes in individual MetS components in over 1.7 million adults [38]. That study reported winter peaks and summer declines in BP and HDL cholesterol levels, whereas other parameters, such as waist circumference, TGs, and fasting glucose, displayed more variable seasonal trends. These findings are consistent with our results, which confirm that metabolic components follow distinct seasonal rhythms. Unlike the Japanese study, which focused only on individual components and did not assess MetS as a defined clinical entity, our analysis comprehensively evaluated both prevalence and severity throughout the year. By leveraging a nationally representative dataset spanning a decade, we were able to stratify participants by sex, age, and socioeconomic factors and assess both the presence and component count of MetS. This approach provides a more integrated understanding of how seasonal factors influence the overall metabolic burden, highlighting important differences in cardiometabolic risk across months and between population subgroups.

This study has several notable strengths. First, the use of the NHIS database enabled the inclusion of a large population-based sample with comprehensive data on lifestyle, anthropometric, laboratory, and medication variables. Second, stratification by sex and month facilitated the exploration of temporal patterns in MetS burden. Third, multivariate analyses accounted for a wide range of confounding variables, including socioeconomic status, health habits, and comorbidities. Despite these strengths, some limitations warrant consideration. First, the cross-sectional nature of the health screening data limits the ability to draw causal inferences regarding seasonal effects. Second, behavioral variables derived from self-reports may be affected by recall or social desirability biases. Finally, the dataset lacked actual environmental variables, such as ambient temperature, daylight duration, and air pollution levels, that could directly reflect seasonal weather trends, and therefore, we could not evaluate whether abrupt weather changes might have amplified the seasonal variation observed. As seasons were defined by calendar months, our findings may not fully capture the impact of year-to-year variability in weather conditions. In addition, information on vitamin D levels was unavailable, which may also have influenced the observed seasonal variation. Although the adjusted ORs observed in this study were modest, these were not unexpected findings that MetS and its components are chronic metabolic indicators that typically change gradually over time and may be less sensitive to short-term environmental fluctuations. In the context of a large-scale population study, even modest effect sizes can represent meaningful differences in population-level cardiometabolic risk. Furthermore, the 8% random sample of NHIS health screening participants was not stratified by month or season, resulting in variation in seasonal sample sizes and demographic composition. While we adjusted for age in multivariable models, residual confounding may remain if the age–season relationship is nonlinear or interacts with other factors. As the primary aim of this study was to evaluate seasonal variation in the prevalence of MetS among adults aged ≥40 years, regardless of age subgroup, age-specific seasonal patterns would be better addressed in a future dedicated study. From clinical and public health perspectives, our findings highlight the importance of accounting for seasonal variation when evaluating MetS and implementing preventive strategies. The increased burden of MetS and its components during winter emphasizes the need for enhanced surveillance, education, and lifestyle interventions during colder months. Seasonally tailored public health messaging and support to promote physical activity and healthy dietary behaviors may be especially beneficial for high-risk populations. In addition, the sex-specific trends observed support the importance of personalized approaches that consider sex-related differences in risk profiles and behaviors. Further research is warranted to clarify the biological mechanisms underlying these seasonal patterns and to evaluate whether seasonally responsive interventions could improve long-term cardiometabolic outcomes. In addition, the TG/HDL-C ratio, a useful marker of insulin resistance and cardiovascular risk, may provide further insight into seasonal and sex-specific patterns when assessed in future studies with extended data access.

## 5. Conclusions

In conclusion, this nationwide study revealed a seasonal variation in the prevalence of MetS and its components among Korean adults, with significant sex-specific differences. The burden of MetS was highest during the colder months, particularly winter, even after adjusting for demographic, behavioral, and socioeconomic factors. Furthermore, men and women exhibited distinct monthly trends. These findings highlight the importance of incorporating seasonal factors into clinical risk assessments and public health strategies targeting MetS. Tailored preventive efforts during high-risk seasons, particularly winter, may contribute to improved metabolic health at the population level.

## Figures and Tables

**Table 1:** Baseline characteristics of men.

Characteristics in Men	All	Spring	Summer	Fall	Winter	*p*-Value
Total N	2,720,212	603,546 (22.2)	610,036 (22.4)	831,135 (30.6)	675,495 (24.8)	
Age (mean ± SD)	55.23 ± 10.86	57.09 ± 11.35	55.02 ± 10.53	54.12 ± 10.39	55.13 ± 11.06	<0.0001
Age group, *n* (%)						<0.0001
<65 years	2,194,295	451,399 (74.8)	500,976 (82.1)	697,860 (84.0)	544,060 (80.5)	
≥65 years	525,917	152,147 (25.2)	109,060 (17.9)	133,275 (16.0)	131,435 (19.5)	
Systolic BP, mmHg	125.71 ± 14.05	125.65 ± 13.91	123.79 ± 13.59	125.8 ± 13.95	127.37 ± 14.48	<0.0001
Diastolic BP, mmHg	78.26 ± 9.79	77.88 ± 9.53	77.03 ± 9.59	78.52 ± 9.77	79.4 ± 10.08	<0.0001
Height, cm	169.29 ± 6.3	168.73 ± 6.34	169.31 ± 6.26	169.63 ± 6.22	169.35 ± 6.36	<0.0001
Weight, kg	70.56 ± 10.68	69.7 ± 10.44	70.11 ± 10.51	71.03 ± 10.66	71.15 ± 10.97	<0.0001
Waist circumference, cm	85.2 ± 8.16	84.98 ± 8.27	84.81 ± 8.04	85.29 ± 8.09	85.62 ± 8.24	<0.0001
Household income, *n* (%)						<0.0001
Quartile 1 (1–5)	433,981	100,793 (16.7)	91,574 (15.0)	124,331 (15.0)	117,283 (17.4)	
Quartile 2 (6–10)	463,897	101,391 (16.8)	100,310 (16.4)	137,006 (16.5)	125,190 (18.5)	
Quartile 3 (11–15)	701,126	150,495 (24.9)	149,886 (24.6)	215,716 (26.0)	185,029 (27.4)	
Quartile 4 (16–20)	1,121,208	250,867 (41.6)	268,266 (44.0)	354,082 (42.6)	247,993 (36.7)	
Area of residence *, *n* (%)						<0.0001
Capital	466,246	96,277 (16.0)	100,975 (16.6)	154,042 (18.5)	114,952 (17.0)	
Metropolitan	715,165	163,740 (27.1)	163,720 (26.8)	218,056 (26.2)	169,649 (25.1)	
Provincial	1,538,801	343,529 (56.9)	345,341 (56.6)	459,037 (55.2)	390,894 (57.9)	
Comorbidities, *n* (%)						
Hypertension	825,796	200,400 (33.2)	183,745 (30.1)	241,024 (29.0)	200,627 (29.7)	<0.0001
Diabetes	324,816	78,196 (13.0)	71,812 (11.8)	94,290 (11.3)	80,518 (11.9)	<0.0001
Dyslipidemia	576,634	138,495 (22.9)	131,561 (21.6)	170,256 (20.5)	136,322 (20.2)	<0.0001
Medication, *n* (%)						
Antihypertensive drugs	909,740	222,171 (36.8)	202,387 (33.2)	264,015 (31.8)	221,167 (32.7)	<0.0001
Glucose-lowering drugs	328,707	79,200 (13.1)	72,659 (11.9)	95,405 (11.5)	81,443 (12.1)	<0.0001
Lipid-lowering drugs	580,882	139,705 (23.1)	132,433 (21.7)	171,378 (20.6)	137,366 (20.3)	<0.0001
Antiplatelet agents	411,646	105,051 (17.4)	90,395 (14.8)	115,138 (13.9)	101,062 (15.0)	<0.0001
Smoking status, *n* (%)						<0.0001
Non-smoker	823,935	191,971 (31.8)	185,135 (30.3)	243,195 (29.3)	203,634 (30.1)	
Past smokers	1,184,790	263,734 (43.7)	273,720 (44.9)	365,948 (44.0)	281,388 (41.7)	
Current smokers	711,487	147,841 (24.5)	151,181 (24.8)	221,992 (26.7)	190,473 (28.2)	
Alcohol, *n* (%)						<0.0001
Low-risk drinking	1,650,535	375,296 (62.2)	371,822 (61.0)	501,618 (60.4)	401,799 (59.5)	
High-risk drinking	1,069,677	228,250 (37.8)	238,214 (39.0)	329,517 (39.6)	273,696 (40.5)	
Physical activity, *n* (%)						<0.0001
Non-regular exercise	1,986,066	431,796 (71.5)	435,799 (71.4)	601,377 (72.4)	517,094 (76.6)	
Regular exercise	734,146	171,750 (28.5)	174,237 (28.6)	229,758 (27.6)	158,401 (23.4)	
BMI, kg/m^2^, *n* (%)						<0.0001
<18.5	46,943	10,776 (1.8)	11,820 (1.9)	13,837 (1.7)	10,510 (1.6)	
18.5–22.9	766,205	178,117 (29.5)	181,015 (29.7)	227,240 (27.3)	179,833 (26.6)	
23.0–24.9	744,835	167,738 (27.8)	169,762 (27.8)	227,812 (27.4)	179,523 (26.6)	
≥25.0	1,162,229	246,915 (40.9)	247,439 (40.6)	362,246 (43.6)	305,629 (45.2)	
Laboratory finding						
Fasting glucose, mg/dL	105.5 ± 28	105.41 ± 27.86	104 ± 26.48	105.11 ± 27.41	107.43 ± 29.99	<0.0001
Total cholesterol, mg/dL	194.54 ± 40.56	192.76 ± 40.28	192.51 ± 39.84	195.99 ± 40.52	196.19 ± 41.35	<0.0001
LDL-C, mg/dL	113.74 ± 39.3	112.99 ± 38.83	112.45 ± 37.36	114.67 ± 40.6	114.43 ± 39.75	<0.0001
HDL-C, mg/dL	52.04 ± 14.73	52.24 ± 15.13	51.06 ± 14.46	52.35 ± 13.69	52.36 ± 15.77	<0.0001
Triglyceride, mg/dL	149.12 ± 103.55	142.92 ± 99.97	150.73 ± 103.6	150.27 ± 103.82	151.79 ± 106.05	<0.0001
eGFR, mL/min/1.73 m^2^	87.94 ± 23.07	87.01 ± 23.76	87.88 ± 22.06	88.21 ± 23.62	88.43 ±22.71	<0.0001
Creatinine, mg/dL	0.99 ± 0.56	0.99 ± 0.63	0.99 ± 0.5	0.99 ± 0.53	0.98 ± 0.57	<0.0001

Seasons were categorized as spring (March–May), summer (June–August), fall (September–November), and winter (December–February). * Capital: Seoul; metropolitan: Busan, Daegu, Incheon, Gwangju, Daejeon, and Ulsan; provincial: Gyeonggi, Gangwon, Chungbuk, Chungnam, Jeonbuk, Jeonnam, Gyeongbuk, Gyeongnam, and Jeju. BP, blood pressure; BMI, body mass index; eGFR, estimated glomerular filtration rate; HDL, high-density lipoprotein; LDL, low-density lipoprotein; SD, standard deviation.

**Table 2:** Baseline characteristics of women.

Characteristics in Women	All	Spring	Summer	Fall	Winter	*p*-Value
Total N	2,787,039	642,450 (23.1)	640,302 (23.0)	785,049 (28.2)	719,238 (25.8)	
Age (mean ± SD)	56.43 ± 11.2	58.87 ± 11.47	56.03 ± 10.77	55.09 ± 10.77	56.06 ± 11.44	<0.0001
Age group, *n* (%)						<0.0001
<65 years	2,170,793	453,147 (75.1)	513,844 (84.2)	643,324 (77.4)	560,478 (83.0)	
≥65 years	616,246	189,303 (31.4)	126,458 (20.7)	141,725 (17.1)	158,760 (23.5)	
Systolic BP, mmHg	122 ± 15.34	122.84 ± 15.39	120.35 ± 14.93	121.7 ± 15.24	123.05 ± 15.63	<0.0001
Diastolic BP, mmHg	74.87 ± 9.82	75.08 ± 9.66	73.87 ± 9.67	74.81 ± 9.82	75.63 ± 9.99	<0.0001
Height, cm	156.14 ± 6.07	155.39 ± 6.11	156.22 ± 5.99	156.58 ± 5.96	156.26 ± 6.17	<0.0001
Weight, kg	57.89 ± 8.89	57.62 ± 8.75	57.46 ± 8.71	58.02 ± 8.89	58.38 ± 9.16	<0.0001
Waist circumference, cm	78.07 ± 9.22	78.62 ± 9.27	77.59 ± 9.2	77.76 ± 9.22	78.36 ± 9.14	<0.0001
Household income, *n* (%)						<0.0001
Quartile 1 (1–5)	729,637	172,744 (28.6)	176,171 (28.9)	204,631 (24.6)	176,091 (26.1)	
Quartile 2 (6–10)	585,854	132,223 (21.9)	143,469 (23.5)	168,126 (20.2)	142,036 (21.0)	
Quartile 3 (11–15)	603,794	139,880 (23.2)	130,861 (21.5)	167,482 (20.2)	165,571 (24.5)	
Quartile 4 (16–20)	867,754	197,603 (32.7)	189,801 (31.1)	244,810 (29.5)	235,540 (34.9)	
Area of residence *, *n* (%)						<0.0001
Capital	506,517	112,862 (18.7)	117,066 (19.2)	151,799 (18.3)	124,790 (18.5)	
Metropolitan	724,334	165,558 (27.4)	168,039 (27.5)	205,753 (24.8)	184,984 (27.4)	
Provincial	1,556,188	364,030 (60.3)	355,197 (58.2)	427,497 (51.4)	409,464 (60.6)	
Comorbidities, *n* (%)						
Hypertension	756,357	205,997 (34.1)	166,718 (27.3)	194,600 (23.4)	189,042 (28.0)	<0.0001
Diabetes	244,982	67,000 (11.1)	53,279 (8.7)	62,108 (7.5)	62,595 (9.3)	<0.0001
Dyslipidemia	637,102	173,555 (28.8)	145,928 (23.9)	166,296 (20.0)	151,323 (22.4)	<0.0001
Medication, *n* (%)						
Antihypertensive drugs	861,299	232,616 (38.5)	190,834 (31.3)	221,842 (26.7)	216,007 (32.0)	<0.0001
Glucose-lowering drugs	249,370	68,265 (11.3)	54,230 (8.9)	63,190 (7.6)	63,685 (9.4)	<0.0001
Lipid-lowering drugs	640,982	174,655 (28.9)	146,751 (24.1)	167,195 (20.1)	152,381 (22.6)	<0.0001
Antiplatelet agents	359,417	102,552 (17.0)	77,984 (12.8)	87,965 (10.6)	90,916 (13.5)	<0.0001
Smoking status, *n* (%)						<0.0001
Non-smoker	2,652,037	613,657 (101.7)	611,544 (100.2)	746,400 (89.8)	680,436 (100.7)	
Past smokers	78,466	16,336 (2.7)	17,037 (2.8)	22,898 (2.8)	22,195 (3.3)	
Current smokers	56,536	12,457 (2.1)	11,721 (1.9)	15,751 (1.9)	16,607 (2.5)	
Alcohol, *n* (%)						<0.0001
Low-risk drinking	2,542,735	594,540 (98.5)	583,440 (95.6)	711,847 (85.6)	652,908 (96.7)	
High-risk drinking	244,304	47,910 (7.9)	56,862 (9.3)	73,202 (8.8)	66,330 (9.8)	
Physical activity, *n* (%)						<0.0001
Non-regular exercise	2,133,132	488,973 (81.0)	479,499 (78.6)	591,224 (71.1)	573,436 (84.9)	
Regular exercise	653,907	153,477 (25.4)	160,803 (26.4)	193,825 (23.3)	145,802 (21.6)	
BMI, kg/m^2,^ *n* (%)						<0.0001
<18.5	89,448	18,913 (3.1)	22,802 (3.7)	26,403 (3.2)	21,330 (3.2)	
18.5–22.9	1,157,374	255,655 (42.4)	278,677 (45.7)	334,566 (40.3)	288,476 (42.7)	
23.0–24.9	650,348	153,761 (25.5)	148,744 (24.4)	181,016 (21.8)	166,827 (24.7)	
≥25.0	889,869	214,121 (35.5)	190,079 (31.2)	243,064 (29.2)	242,605 (35.9)	
Laboratory finding						
Fasting glucose, mg/dL	99.37 ± 22.29	100.26 ± 22.97	98.46 ± 21.23	98.66 ± 21.45	100.16 ± 23.39	<0.0001
Total cholesterol, mg/dL	200.11 ± 40.51	199.73 ± 41.9	199.52 ± 40.06	200.57 ± 39.62	200.48 ± 40.59	<0.0001
LDL-C, mg/dL	117.73 ± 38.97	117.82 ± 39.55	117.75 ± 39.73	117.75 ± 37.66	117.62 ± 39.18	0.0179
HDL-C, mg/dL	59.68 ± 15.98	59.31 ± 16.58	58.9 ± 15.66	60.56 ± 15.2	59.75 ± 16.48	<0.0001
Triglyceride, mg/dL	113.99 ± 68.35	113.38 ± 67.66	114.96 ± 68.6	111.51 ± 66.4	116.37 ± 70.69	<0.0001
eGFR, mL/min/1.73 m^2^	90.05 ± 24.65	88.59 ± 24.91	89.78 ± 24.07	90.73 ± 24.73	90.78 ±24.8	<0.0001
Creatinine, mg/dL	0.75 ± 0.55	0.76 ± 0.61	0.75 ± 0.55	0.75 ± 0.52	0.75 ± 0.5	<0.0001

Seasons were categorized as spring (March–May), summer (June–August), fall (September–November), and winter (December–February). * Capital: Seoul; metropolitan: Busan, Daegu, Incheon, Gwangju, Daejeon, and Ulsan; provincial: Gyeonggi, Gangwon, Chungbuk, Chungnam, Jeonbuk, Jeonnam, Gyeongbuk, Gyeongnam, and Jeju. BP, blood pressure; BMI, body mass index; eGFR, estimated glomerular filtration rate; HDL, high-density lipoprotein; LDL, low-density lipoprotein; SD, standard deviation.

**Table 3:** Prevalence of metabolic syndrome and its components by season.

Men	All	Spring	Summer	Fall	Winter	*p*-Value
Total N, *n* (%)	2,720,212	603,546 (22.2)	610,036 (22.4)	831,135 (30.6)	675,495 (24.8)	
Metabolic syndrome, *n* (%)	861,740	184,522 (30.6)	184,232 (30.2)	260,113 (31.3)	232,873 (34.5)	<0.0001
Component of metabolic syndrome, *n* (%)						<0.0001
0	377,068	83,114 (13.8)	93,657 (15.4)	118,430 (14.2)	81,867 (12.1)	
1	699,016	158,976 (26.3)	160,469 (26.3)	214,664 (25.8)	164,907 (24.4)	
2	782,388	176,934 (29.3)	171,678 (28.1)	237,928 (28.6)	195,848 (29.0)	
3	557,543	121,129 (20.1)	118,839 (19.5)	168,888 (20.3)	148,687 (22.0)	
4	251,785	52,436 (8.7)	53,769 (8.8)	75,821 (9.1)	69,759 (10.3)	
5	52,412	10,957 (1.8)	11,624 (1.9)	15,404 (1.9)	14,427 (2.1)	
Elevated waist circumference, *n* (%)	753,413	162,069 (26.9)	158,444 (26.0)	231,999 (27.9)	200,901 (29.7)	<0.0001
High fasting glucose, *n* (%)	1,370,867	305,000 (50.5)	291,490 (48.7)	413,045 (49.7)	361,332 (53.5)	<0.0001
High blood pressure, *n* (%)	1,650,742	375,648 (62.3)	347,978 (57.0)	498,872 (60.0)	428,244 (63.4)	<0.0001
Hypertriglyceridemia, *n* (%)	1,009,035	205,430 (34.0)	230,791 (37.8)	313,058 (37.7)	259,756 (38.5)	<0.0001
Low HDL cholesterol, *n* (%)	421,564	92,613 (15.3)	104,835 (17.0)	120,514 (14.5)	103,602 (15.3)	<0.0001
Women	All	Spring	Summer	Fall	Winter	*p*-value
Total N, *n* (%)	2,787,039	642,450 (23.1)	640,302 (23.0)	785,049 (28.2)	719,238 (25.8)	
Metabolic syndrome, *n* (%)	655,161	164,044 (25.5)	143,312 (22.4)	168,826 (21.5)	178,979 (24.9)	<0.0001
Component of metabolic syndrome, *n* (%)						<0.0001
0	717,290	147,263 (22.9)	173,942 (27.2)	219,346 (27.9)	176,739 (24.6)	
1	781,082	177,076 (27.6)	181,226 (28.3)	224,425 (28.6)	198,355 (27.6)	
2	633,506	154,067 (24.0)	141,822 (22.1)	172,452 (22.0)	165,165 (23.0)	
3	408,211	101,769 (15.8)	89,362 (14.0)	106,836 (13.6)	110,244 (15.3)	
4	192,302	48,244 (7.5)	42,258 (6.6)	48,478 (6.2)	53,322 (7.4)	
5	54,648	14,031 (2.2)	11,692 (1.8)	13,512 (1.7)	15,413 (2.1)	
Elevated waist circumference, *n* (%)	621,870	155,471 (24.2)	132,149 (20.6)	166,654 (21.2)	167,596 (23.3)	<0.0001
High fasting glucose, *n* (%)	1,020,353	250,616 (39.0)	222,140 (34.7)	274,613 (35.0)	272,984 (38.0)	<0.0001
High blood pressure, *n* (%)	1,376,848	345,732 (53.8)	296,026 (46.2)	371,549 (47.3)	363,541 (50.6)	<0.0001
Hypertriglyceridemia, *n* (%)	580,333	131,442 (20.5)	135,825 (21.2)	153,885 (19.6)	159,181 (22.1)	<0.0001
Low HDL cholesterol, *n* (%)	715,771	170,387 (26.5)	174,308 (27.2)	184,608 (23.5)	186,468 (25.9)	<0.0001

Seasons were categorized as spring (March–May), summer (June–August), fall (September–November), and winter (December–February). Component of metabolic syndrome indicates the number of criteria met (0–5) based on the harmonized definition: (1) abdominal obesity (waist circumference ≥ 90 cm in men or ≥85 cm in women), (2) elevated triglycerides (≥150 mg/dL), (3) low HDL-C (<40 mg/dL in men or <50 mg/dL in women), (4) elevated blood pressure (systolic ≥ 130 mmHg, diastolic ≥ 85 mmHg, or use of antihypertensive medication), and (5) elevated fasting glucose (≥100 mg/dL or use of glucose-lowering medication). Prevalence of each individual component is also shown separately in the table. HDL, high-density lipoprotein.

## Data Availability

This study used data from the NHID of Korea. The data are not publicly available but can be accessed through the NHID data request process for approved research purposes (https://nhiss.nhis.or.kr (accessed on 10 July 2025)).

## Supplementary Materials

The following supporting information can be downloaded at https://www.mdpi.com/article/10.3390/jcm14175968/s1. Table S1: Codes used to define comorbidities and medication history; Table S2: Effect of season on metabolic syndrome prevalence by sex.

## Abbreviations

The following abbreviations are used in this manuscript:

MetS	Metabolic syndrome
BP	Blood pressure
NHIS	National Health Insurance Service
BMI	Body mass index
TG	Triglyceride
HDL-C	High-density lipoprotein cholesterol
OR	Odds ratio
95% CI	95% confidence interval
eGFR	Estimated glomerular filtration rate
