# Supplementary material for: Sex Differences in Seasonal Variation in Metabolic Syndrome and Its Components: A 10-Year National Health Screening Study
Source: J Clin Med. 2025 Aug 23;14(17):5968. doi: 10.3390/jcm14175968 (PMC12429781; doi:10.3390/jcm14175968)
Supplement: Supplementary file 1 [file jcm-14-05968-s001.zip › jcm-3812433-supplementary.pdf]

Supplementary Materials

Supplemental Table S1. Codes used to define comorbidities and medication history

Diagnosis	Definition
Hypertension	Defined as ICD-10 codes I10–13 and I15 and prescription of antihypertensive agents
Diabetes mellitus	Defined as ICD-10 codes E10–14 and prescription of antidiabetic agents
Dyslipidemia	Defined as ICD-10 codes E78 and prescription of lipid-lowering agents
**Medications**	
Statins	Rosuvastatin, atorvastatin, simvastatin, pitavastatin, pravastatin, fluvastatin, lovastatin
Other lipid-lowering drugs	evolocumab, alirocumab, ezetimibe, fenofibrate, bezafibrate, ciprofibrate, etofibrate, gemfibrozil, omega-3, nicotinic acid, acipimox
Antihypertensive agents	Amlodipine, nifedipine, nilvadipine, nimodipine, barnidipine, benidipine, cilnidipine, felodipine, isradipine, lacidipine, lercanidipine, manidipine, moxonidine, nicardipine, nitrendipine, nisoldipine, efonidipine, verapamil, diltiazem, azilsartan, candesartan, eprosartan, fimasartan, irbesartan, losartan, olmesartan, telmisartan, valsartan, alacepril, benazepril, captopril, cilazapril, enalapril, fosinopril, imidapril, lisinopril, moexipril, perindopril, ramipril, temocapril, zofenopril, carvedilol, atenolol, nebivolol, bisoprolol, metoprolol, amosulalol, arotinolol, betaxolol, bevantolol, celiprolol, labetalol, nadolol, propranolol, hydrochlorothiazide, chlorthalidone, tripamide, spironolactone, amiloride, furosemide, azosemide, torasemide, xipamide, metolazone, prazosin, cicletanine, clonidine, minoxidil, cadralazine
Antidiabetic agents	Metformin, gemigliptin, sitagliptin, vildagliptin, saxagliptin, linagliptin, teneligliptin, alogliptin, anagliptin, evogliptin, dapagliflozin, empagliflozin, ipragliflozin, ertugliflozin, enavogliflozin, acarbose, miglitol, voglibose, glibenclamide, gliclazide, glimepiride, glipizide, gliquidone, rosiglitazone, pioglitazone, lobeglitazone, repaglinide, nateglinide, mitiglinide, exenatide, lixisenatide, dulaglutide, albiglutide, insulin
Antiplatelet agents	Aspirin, clopidogrel, prasugrel, ticagrelor, cilostazol, dipyridamole, ticlopidine

ICD-10, International Classification of Diseases-10th revision.

Supplemental Table S2. Effect of season on metabolic syndrome prevalence by sex

						Model 1*		Model 2†		Model 3‡	
Men	Season	Total	Events	OR (95% CI)	*p*-value	OR (95% CI)	*p* -value	OR (95% CI)	*p* -value	OR (95% CI)	*p* -value
Metabolic syndrome	Spring	209,181	70,540	1 (reference)		1 (reference)		1 (reference)		1 (reference)	
	Summer	229,122	75,419	0.964 (0.952–0.977)	<.0001	0.987 (0.974–0.999)	0.0381	0.989 (0.976–1.001)	0.0836	0.991 (0.978–1.003)	0.1525
	Fall	299,270	101,545	1.009 (0.998–1.021)	0.1213	1.043 (1.03–1.055)	<.0001	1.043 (1.031–1.056)	<.0001	1.045 (1.033–1.058)	<.0001
	Winter	258,941	95,288	1.144 (1.131–1.158)	<.0001	1.176 (1.162–1.191)	<.0001	1.161 (1.147–1.176)	<.0001	1.159 (1.145–1.173)	<.0001
≥ 1 Component	Spring	209,181	185,500	1 (reference)		1 (reference)		1 (reference)		1 (reference)	
	Summer	229,122	199,082	0.846 (0.831–0.862)	<.0001	0.918 (0.902–0.936)	<.0001	0.919 (0.902–0.936)	<.0001	0.923 (0.906–0.941)	<.0001
	Fall	299,270	262,499	0.911 (0.896–0.927)	<.0001	1.029 (1.011–1.047)	0.0014	1.028 (1.01–1.046)	0.0021	1.033 (1.015–1.051)	0.0004
	Winter	258,941	231,346	1.07 (1.051–1.09)	<.0001	1.194 (1.172–1.216)	<.0001	1.177 (1.155–1.199)	<.0001	1.172 (1.15–1.194)	<.0001
≥ 2 Components	Spring	209,181	134,123	1 (reference)		1 (reference)		1 (reference)		1 (reference)	
	Summer	229,122	141,787	0.909 (0.897–0.92)	<.0001	0.955 (0.943–0.966)	<.0001	0.956 (0.944–0.968)	<.0001	0.958 (0.946–0.97)	<.0001
	Fall	299,270	189,538	0.967 (0.955–0.978)	<.0001	1.038 (1.026–1.05)	<.0001	1.038 (1.026–1.05)	<.0001	1.041 (1.029–1.054)	<.0001
	Winter	258,941	171,286	1.094 (1.08–1.107)	<.0001	1.164 (1.15–1.178)	<.0001	1.15 (1.136–1.164)	<.0001	1.147 (1.133–1.161)	<.0001
≥ 4 Components	Spring	209,181	24,929	1 (reference)		1 (reference)		1 (reference)		1 (reference)	
	Summer	229,122	27,312	1.000 (0.982–1.019)	0.9767	1.012 (0.994–1.031)	0.1855	1.016 (0.998–1.035)	0.0869	1.018 (0.999–1.037)	0.0586
	Fall	299,270	36,287	1.020 (1.002–	0.0251	1.037 (1.02–	<.0001	1.038 (1.021–	<.0001	1.040 (1.022–	<.0001

				1.038)		1.055)		1.056)		1.058)	
	Winter	258,941	35,051	1.157 (1.137–1.177)	<.0001	1.174 (1.154–1.195)	<.0001	1.158 (1.138–1.178)	<.0001	1.155 (1.135–1.176)	<.0001
≥ 5 Components	Spring	209,181	4,321	1 (reference)		1 (reference)		1 (reference)		1 (reference)	
	Summer	229,122	4,892	1.034 (0.992–1.078)	0.1095	1.063 (1.02–1.108)	0.0038	1.070 (1.026–1.115)	0.0015	1.073 (1.029–1.118)	0.0009
	Fall	299,270	6,220	1.006 (0.968–1.047)	0.7542	1.046 (1.005–1.088)	0.0266	1.047 (1.007–1.09)	0.0214	1.051 (1.01–1.093)	0.0136
	Winter	258,941	6,188	1.161 (1.116–1.207)	<.0001	1.199 (1.152–1.247)	<.0001	1.185 (1.139–1.232)	<.0001	1.183 (1.137–1.23)	<.0001

Women	Season	Total	Events	OR (95% CI)	p-value	OR (95% CI)	p-value	OR (95% CI)	p-value	OR (95% CI)	p-value
Metabolic syndrome	Spring	231,749	65,080	1 (reference)		1 (reference)		1 (reference)		1 (reference)	
	Summer	249,255	61,936	0.847 (0.836–0.858)	<.0001	0.997 (0.984–1.011)	0.6757	1.002 (0.989–1.016)	0.7596	1.003 (0.99–1.017)	0.6211
	Fall	306,186	72,780	0.799 (0.789–0.808)	<.0001	0.996 (0.983–1.009)	0.5547	0.999 (0.987–1.013)	0.9365	1.005 (0.992–1.018)	0.4232
	Winter	274,193	73,480	0.938 (0.926–0.949)	<.0001	1.14 (1.125–1.155)	<.0001	1.13 (1.116–1.145)	<.0001	1.134 (1.119–1.149)	<.0001
≥ 1 Component	Spring	231,749	183,729	1 (reference)		1 (reference)		1 (reference)		1 (reference)	
	Summer	249,255	186,887	0.783 (0.773–0.794)	<.0001	0.947 (0.933–0.961)	<.0001	0.951 (0.937–0.965)	<.0001	0.955 (0.941–0.969)	<.0001
	Fall	306,186	227,671	0.758 (0.748–0.768)	<.0001	1.005 (0.991–1.019)	0.4798	1.008 (0.994–1.022)	0.2724	1.019 (1.005–1.033)	0.0095
	Winter	274,193	210,947	0.872 (0.86–0.884)	<.0001	1.147 (1.13–1.164)	<.0001	1.139 (1.123–1.156)	<.0001	1.148 (1.132–1.165)	<.0001
≥ 2 Components	Spring	231,749	122,676	1 (reference)		1 (reference)		1 (reference)		1 (reference)	
	Summer	249,255	119,148	0.814 (0.805–0.824)	<.0001	0.975 (0.963–0.987)	<.0001	0.98 (0.968–0.992)	0.0011	0.982 (0.97–0.994)	0.0034

	Fall	306,186	142,869	0.778 (0.77–0.786)	<.0001	1.002 (0.99–1.013)	0.7819	1.005 (0.993–1.017)	0.4343	1.012 (1.001–1.024)	0.0397
	Winter	274,193	138,015	0.901 (0.891–0.911)	<.0001	1.142 (1.128–1.156)	<.0001	1.134 (1.12–1.147)	<.0001	1.14 (1.126–1.153)	<.0001
≥ 4 Components	Spring	231,749	25,098	1 (reference)		1 (reference)		1 (reference)		1 (reference)	
	Summer	249,255	23,915	0.874 (0.858–0.89)	<.0001	1.018 (0.999–1.038)	0.0617	1.024 (1.005–1.044)	0.0145	1.025 (1.006–1.045)	0.0106
	Fall	306,186	27,397	0.809 (0.795–0.824)	<.0001	0.993 (0.975–1.012)	0.4714	0.997 (0.979–1.016)	0.7635	1.002 (0.984–1.021)	0.8301
	Winter	274,193	28,507	0.955 (0.938–0.973)	<.0001	1.135 (1.114–1.156)	<.0001	1.125 (1.104–1.146)	<.0001	1.126 (1.106–1.147)	<.0001
≥ 5 Components	Spring	231,749	5,845	1 (reference)		1 (reference)		1 (reference)		1 (reference)	
	Summer	249,255	5,305	0.84 (0.809–0.873)	<.0001	0.984 (0.948–1.022)	0.4168	0.99 (0.954–1.029)	0.6211	0.991 (0.954–1.03)	0.6483
	Fall	306,186	6,168	0.795 (0.766–0.824)	<.0001	0.983 (0.947–1.019)	0.3475	0.987 (0.951–1.024)	0.4839	0.991 (0.955–1.028)	0.6233
	Winter	274,193	6,573	0.949 (0.916–0.984)	0.0042	1.128 (1.089–1.17)	<.0001	1.118 (1.078–1.159)	<.0001	1.12 (1.08–1.161)	<.0001

*Model 1 includes adjustments for age.

†Model 2 includes adjustments for age, smoking status, alcohol consumption, and physical activity (lifestyle model).

‡Model 3 includes adjustments for age, smoking status, alcohol consumption, physical activity, household income, and area of residence (socioeconomic model).
